# Community Needs Assessment Collaboration Following the July 2022 Flooding in Eastern Kentucky

**DOI:** 10.13023/jah.0601.11

**Published:** 2024-09-01

**Authors:** Melissa Slone, Frances Feltner, William M. Baker, Anthony S. Lockard, Angela Raleigh

**Affiliations:** Kentucky River District Health Department; University of Kentucky Center of Excellence in Rural Health; University of Kentucky Center of Excellence in Rural Health; Kentucky River District Health Department; Breathitt County Health Department

**Keywords:** Appalachia, assessment tool, community coalitions, rural

## Abstract

Rapidly rising waters due to flash floods and thunderstorms on the night of July 27, 2022, resulted in hundreds of water rescues throughout 14 rural Appalachian Kentucky counties. Lives were lost, thousands were injured, homes and property were damaged or destroyed, and many roadways were unpassable. Community partners serving these counties collaborated to design and conduct an assessment to gain a better understanding of the needs of individuals residing in certain remote sections within the communities. The assessment, conducted three months after the flood, collected information regarding flooding impact on housing, physical and behavioral health, transportation, work, and finances.

On July 25, 2022, and continuing until July 30, 2022, communities in Leslie, Owsley, Breathitt, Perry, Knott and Letcher Counties saw rainfall totals reach upwards of 14–16 inches.^1^ The flooding resulted in hundreds of bridges being damaged or destroyed and roads being washed away or blocked by debris.^2^ Many people were injured or killed, businesses were forced to close, and homes and property were lost or damaged. To get a better understanding of the needs of those impacted, four groups (the Kentucky River Health Consortium, Kentucky River District Health Department, Breathitt County Health Department, Kentucky Homeplace, and the University of Kentucky Center of Excellence in Rural Health) joined efforts to design and implement the Kentucky River Flood Impact Assessment. A pilot test of this door-to-door assessment was conducted in one of the counties where flooding was confined to a specific area. The pilot feedback was used to finalize the assessment before conducting in other counties and placing on social media.

Volunteers were engaged to conduct the door-to-door assessments in the four counties (Knott, Letcher, Breathitt, and Perry) most impacted by the flood. The assessment was also shared via Facebook. As a result of both formats, 87 responses were collected. The majority (87%) reported living in a single-family home, with the number of individuals living in the home ranging from one to eight; 35.21% reported having someone with a disability in their home. The majority (72.5%) reported their homes were either in need of major repairs or damaged beyond repair/structure no longer there. Of those in their home, 43.59% stated they did not feel safe. When asked about the barriers to leaving an unsafe house or property, 38% stated fear of theft and 48% responded “other.” Other responses included “hard to leave,” “have to stay to get things done [sic] we both work day shift,” “doesn’t pay rent and can’t afford to,” “lots of financial value within the house,” and “have nowhere left to go, this is our property.”

In addition to long-term needs, these assessments also identified many immediate basic needs of individuals. Beds and bedding items were most identified as a need (43%). Other needed items were heating and air conditioning (15.82%); gas/propane (13.29%); refrigeration (13.29%); electricity (10.76%); water for drinking (9.49%); water for bathing (8.23%); a functioning toilet (8.23%); food (8.23%); and a phone (5.06%). Assistance with completing Federal Emergency Management Agency (FEMA) forms and securing copies of important documents (e.g., social security cards and birth certificates) were also identified as needs.

Many respondents reported experiencing physical health symptoms following the flood. These included a cough (21.05%); rash (15.79%); red/itchy eyes (15.79%); nausea (14.04%); diarrhea (14.04%); wounds (10.52%); and fevers (8.77%). Participants also reported worsening of allergies (28.33%); asthma/COPD (20%); hypertension (15%); diabetes (13.33%); and mental health conditions (11.67%). Mental health concerns also increased following the flooding. When asked how often since the flooding they had felt down, depressed, or hopeless, 23.35% of respondents reported several days, 16.90% more than half the days, and 39.44% nearly every day. When asked how often they had felt nervous, anxious, or on edge, 19.72% reported several days, 12.68% more than half the days, and 46.48% nearly every day. Over 60% reported they had been unable to stop or control worrying more than half the days or nearly every day since the flood.

Over half of respondents reported loss of eyeglasses (46.81%); dentures (21.28%); hearing aids (4.26%); and durable medical equipment (27.66%). Over 30% reported their job or income was impacted by the flood. Damage to the work location (20%) and “other” (60%) were the top responses. Comments for “other” included “took time off to work on home and got fired,” “dealing with FEMA and trying to find a home,” “had to move,” “lost business,” “husband had to miss due to a flood injury,” “mentally unable,” and “we lost our school.”

As assessments were completed and entered into the database, individuals with immediate needs were referred to the Kentucky Homeplace Community Health Worker in their county. Long-term needs were shared with each county’s long-term flood recovery group. Overall, most in-person assessment participants wanted someone to listen to their story. They appreciated the offer to refer for assistance but thanked volunteers the most for simply checking on them and listening.
